# Editorial: Metabotropic Glutamate Receptors and Neurological/Psychiatric Disorders

**DOI:** 10.3389/fnmol.2019.00067

**Published:** 2019-03-22

**Authors:** Enza Palazzo, Volker Neugebauer, Sabatino Maione

**Affiliations:** ^1^Department of Experimental Medicine, University of Campania “L. Vanvitelli”, Naples, Italy; ^2^Department of Pharmacology and Neuroscience, School of Medicine, Texas Tech University Health Sciences Center, Lubbock, TX, United States; ^3^Garrison Institute on Aging, Texas Tech University Health Sciences Center, Lubbock, TX, United States

**Keywords:** mGluRs, neurologcial disorders, psychiatric disorders, neuroinflammation, pharmacological manipulation

Neurological and psychiatric disorders significantly impact the quality of life, present devastating symptoms, cause severe disabilities, and are becoming more and more common. No efficient therapies are available at the moment. Thus, revealing novel targets and consequent interventions appears fundamental. Although the term “neurological and psychiatric disorders” encompasses a large number of heterogeneous illnesses, common underlying mechanisms are emerging. Alterations in adult neurogenesis and development of neuroinflammation appear indeed shared hallmarks of both neurodegenerative and psychiatric illnesses. Neurogenesis and neuroinflammation are in turn associated with glutamate, whose excessive or prolonged exposure leads to death of neurons, a key mechanism underlying neurodegenerative diseases. In addition, glutamate hyper- or dys-function has been implicated in the neuropathology of a number of other disorders. Therefore, strategies to control glutamate functions may have therapeutic potential.

Metabotropic glutamate receptors (mGluRs) provide an exciting toolbox to modulate neuronal responses to the pathological actions of glutamate; they are broadly distributed throughout the central nervous system and act on sites and synapses that are considered critical for certain neuropsychiatric disorders. As a consequence, mGluR manipulations produce a variety of responses depending on their different localization on synaptic elements, cell population (neurons or glia), and peripheral or central nervous system sites. In this Research Topic, we aimed for a state of the art summary of our current knowledge about the effect of mGluR manipulations in animal models of neurological and psychiatric diseases. To do so we were fortunate to recruit several renowned experts in the field to share their points of view on the most influential discoveries in this exciting area of research, with particular emphasis on the role and benefits of positive and negative allosteric modulators for mGluR as potential interventions in neurological and psychiatric disorders.

The role of mGluRs in chronic pain is addressed in three contributions. The article by Pereira and Goudet describes the role of mGluRs in pain control and therapeutic strategies aimed at their modulation throughout the pain neuraxis. Mazzitelli et al. review the potential usefulness of group II mGluRs for pain control because of their generally inhibitory synaptic and cellular action and their location on peripheral, spinal, and supraspinal neural elements involved in pain processing and pain modulation. The involvement of the mGluR5 subtype in medial prefrontal cortex (mPFC) activity changes associated with pain and depression is addressed by Chung et al. The mPFC represents the executive center for reciprocal interactions between pain and depression, but the role of mGluR5 in these interactions is still controversial.

The involvement of mGluR5 in social behavior and anxiety, however, has been investigated in detail by Ramos-Prats et al. using mGluR5 negative allosteric modulators and mGluR5 knockout strategies. Continuing with the spectrum of neuropsychiatric disorders, Matrisciano et al. addresses the role of mGluR2/3 in a mouse model of prenatal stress-induced schizophrenia, where activation of mGluR2/3 reverted the molecular and behavioral anomalies associated with the early phase of schizophrenia. The therapeutic potential of mGluR7 in chronic psychosis is the focus of the article by Cieślik et al. that describes studies to address the pharmacokinetic and pharmacodynamic properties and *in vivo* effects of negative allosteric modulators for mGluR7 in animal models of schizophrenia. The authors conclude that the mGluR7 receptor is a putative target for novel antipsychotic therapies.

The therapeutic potential of mGluR7 is not limited to psychotic disorders but extends to neurodevelopmental disorders, including intellectual, learning, communication, and motor disabilities, autism spectrum, and attention-hyperactivity disorders. Fisher et al. provide evidence that mutation or decreased expression of mGluR7 is associated with symptoms overlapping those of neurodevelopmental disorders; conversely, positive modulation of mGluR7 proved beneficial in preclinical studies. Vergassola et al. studied the interaction between mGluR1 and GABA_B_ receptor on GABAergic and glutamatergic cortical terminals. This previously unknown receptor-receptor interaction, which occurs at pre-synaptic level, opens up an unexplored panorama to be exploited for the indirect modulation of inhibitory and excitatory drives in opposite directions, which may have protective and beneficial effects in all neuropsychiatric pathologies associated with “hyperglutamatergism.” The role of mGluRs in neuroinflammation is addressed in two review articles that provide the reader with the basics needed to appreciate the seemingly unlimited potential of mGluRs in CNS diseases. Spampinato et al. focuses on mGluRs expressed on glial cells, microglia, astrocytes, and oligondendrocytes, all important players in the development and maintenance of neuroinflammation. Crupi et al. describe the role of each mGluR group and subtype in neuroinflammation-dependent neurological disorders.

And so we hope that this Research Topic highlighting the unique multi-faceted functions of mGluRs in peripheral and central nervous system disorders will make an original contribution to the field that is useful to basic scientists and clinicians interested in understanding CNS mechanisms of neurological and psychiatric disorders and developing new therapies.

## Author Contributions

EP, VN, and SM contributed and collaborated profitably in the realization and organization of the Research Topic and in the drafting of the editorial article.

### Conflict of Interest Statement

The authors declare that the research was conducted in the absence of any commercial or financial relationships that could be construed as a potential conflict of interest.

